# Supplementary figures and images for: Perceived gender equitable norms and previous tuberculosis testing in Malawi: A secondary analysis of a cluster-based prevalence survey
Source: PLOS Glob Public Health. 2026 Feb 12;6(2):e0004620. doi: 10.1371/journal.pgph.0004620 (PMC12900314; doi:10.1371/journal.pgph.0004620)

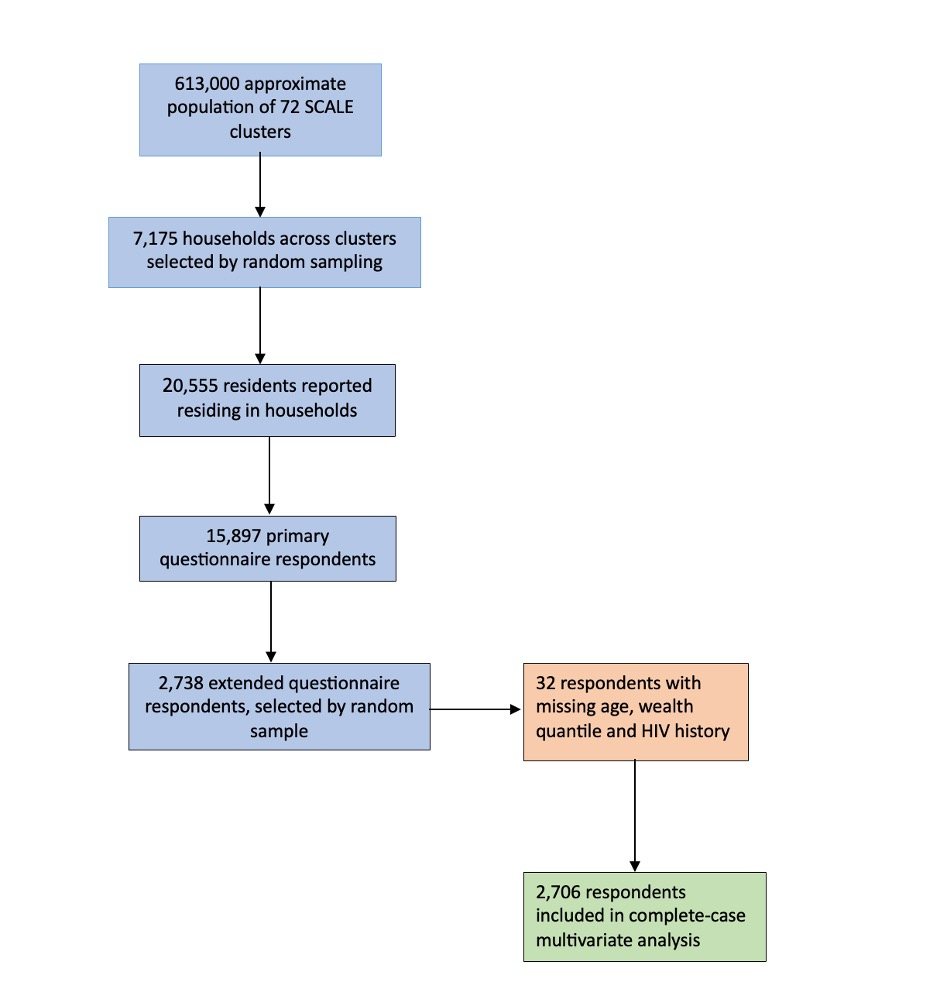

Supplement: S1 Fig — (TIFF) [file pgph.0004620.s001.tiff]

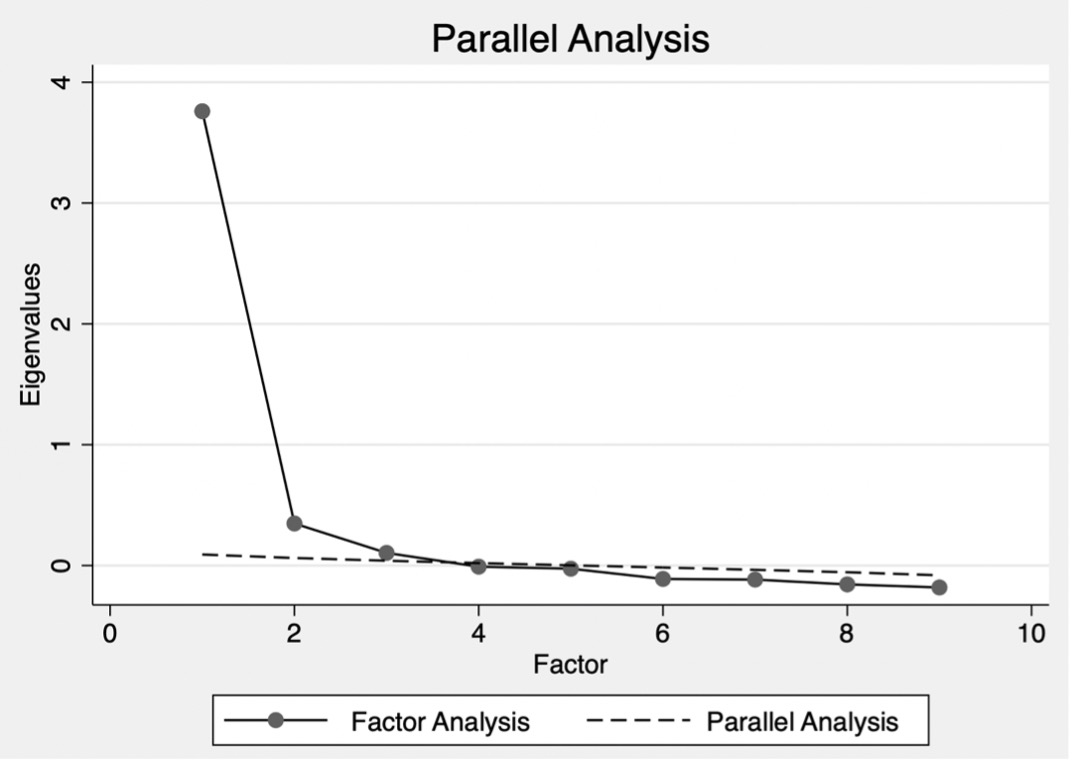

Supplement: S2 Fig — (TIFF) [file pgph.0004620.s002.tiff]
