# Supplementary material for: Perceived gender equitable norms and previous tuberculosis testing in Malawi: A secondary analysis of a cluster-based prevalence survey
Source: PLOS Glob Public Health. 2026 Feb 12;6(2):e0004620. doi: 10.1371/journal.pgph.0004620 (PMC12900314; doi:10.1371/journal.pgph.0004620)
Supplement: S1 Table — (DOCX) [file pgph.0004620.s003.docx]

**S1 Table: Characteristics of participants by HIV status**

| **Characteristic** | **Total (%) (N= 2,621)** | **HIV status** | | **OR (95% CI)** | **Chi-squared p-value** |
| --- | --- | --- | --- | --- | --- |
|  |  | **Positive (%)**  **N=329**  **(12.55%)** | **Negative (%)**  **N=2,292**  **(87.45%)** |  |  |
| ***DEMOGRAPHIC CHARACTERISTICS*** | | | | | |
| ***Sex*** | | | | | |
| Female | 1614 | 248 (15.37) | 1366 (84.63) | 1.00 (Reference) | <0.001 |
| Male | 1007 | 81 (8.04) | 926 (91.96) | 0.48 (0.37-0.63) |  |
| ***Age Group (years)*** | | | | | |
| 18-24 | 969 | 28 (2.89) | 941 (97.11) | 1.00 (Reference) |  |
| 25-34 | 744 | 72 (9.68) | 672 (90.32) | 3.60 (2.30-5.63) | <0.001 |
| 35-44 | 457 | 113 (24.73) | 344 (75.27) | 11.04 (7.17-17.00) | <0.001 |
| 45-54 | 222 | 73 (32.88) | 149 (67.12) | 16.47 (10.30-26.31) | <0.001 |
| ≥55 | 226 | 42 (18.58) | 184 (81.42) | 7.67 (4.64-12.69) | <0.001 |
| ***SOCIOECONOMIC CHARACTERISTICS*** | | | | | |
| ***Self-reported Wealth Index*** | | | | | |
| Step 1 (poorest) | 179 | 36 (20.11) | 143 (79.89) | 1.00 (Reference) | ---------- |
| Step 2 | 622 | 79 (12.7) | 543 (87.3) | 0.58 (0.37-0.89) | 0.013 |
| Step 3 | 1164 | 147 (12.63) | 1017 (87.37) | 0.57 (0.38-0.86) | 0.007 |
| Step 4 | 517 | 53 (10.25) | 464 (89.75) | 0.45 (0.29-0.72) | 0.001 |
| Step 5 | 85 | 9 (10.59) | 76 (89.41) | 0.47 (0.22-1.03) | 0.059 |
| Step 6 (richest) | 26 | 3 (11.54) | 23 (88.46) | 0.52 (0.14-1.82) | 0.305 |
| ***Education*** | | | | | |
| Never attended School or not completed primary | 487 | 88 (18.07) | 399 (81.93) | 1.00 (Reference) | ----------- |
| Primary school or Junior certificate | 1181 | 169 (14.31) | 1012 (85.69) | 0.76 (0.57-1.00) | 0.054 |
| Secondary and Higher Education | 953 | 72 (7.56) | 881 (92.44) | 0.37 (0.18-0.28) | <0.001 |
| **Literacy (Able to read a newspaper or letter in English or Chichewa)** | | | | | |
| Yes | 2416 | 297 (12.29) | 2119 (87.71) | 1.00 (Reference) | 0.170 |
| No | 205 | 31 (15.12) | 173 (84.39) | 1.31 (0.89-1.96) |  |
| **Employment** | | | | | |
| Paid Employee (including piece work and domestic work) | 516 | 74 (14.34) | 442 (85.66) | 1.00 (Reference) | ------------ |
| Self-Employed | 663 | 111 (16.74) | 552 (83.26) | 1.20 (0.87-1.65) | 0.261 |
| Unemployed | 1066 | 138 (12.95) | 928 (87.05) | 0.89 (0.66-1.20) | 0.445 |
| Student and other | 376 | 6 (1.6) | 370 (98.4) | 0.10 (0.04-0.23) | <0.001 |
