# Supplementary material for: Perceived gender equitable norms and previous tuberculosis testing in Malawi: A secondary analysis of a cluster-based prevalence survey
Source: PLOS Glob Public Health. 2026 Feb 12;6(2):e0004620. doi: 10.1371/journal.pgph.0004620 (PMC12900314; doi:10.1371/journal.pgph.0004620)
Supplement: S2 Table — (DOCX) [file pgph.0004620.s004.docx]

**S2 Table: GEMS Scale Questions and Responses, by Sex**

| **Item Number** | **Question*** | **Sex** | **Strongly Agree**  **n (%)** | **Somewhat Agree**  **n (%)** | **Do Not Agree**  **n (%)** |
| --- | --- | --- | --- | --- | --- |
| 1 | “Changing **diapers**, giving kids a bath & feeding kids are mother's responsibility.” | **Female** | 1244 (74.76) | 172 (10.34) | 248 (14.9) |
|  |  | **Male** | 792 (73.74) | 122 (11.36) | 160 (14.9) |
| 2 | “A man should have the final word about **decisions** in his home.” | **Female** | 1,089 (65.44) | 231 (13.88) | 344 (20.67) |
|  |  | **Male** | 741 (68.99) | 154 (14.34) | 179 (16.67) |
| 3 | “There are times when a woman deserves to be **beaten**.” | **Female** | 612 (36.78) | 236 (14.18) | 816 (49.04) |
|  |  | **Male** | 436 (40.60) | 191 (17.78) | 447 (41.62) |
| 4 | “Men need **sex more** than women do.” | **Female** | 936 (56.25) | 297 (17.85) | 431 (25.90) |
|  |  | **Male** | 567 (52.79) | 238 (22.16) | 269 (25.05) |
| 5 | “Men **don't talk** about sex, they just do it.” | **Female** | 894 (53.75) | 296 (17.79) | 474 (28.49) |
|  |  | **Male** | 583 (54.28) | 210 (19.55) | 281 (26.16) |
| 6 | “Men are **always ready** to have sex.” | **Female** | 930 (55.89) | 300 (18.03) | 434 (26.08) |
|  |  | **Male** | 598 (55.68) | 213 (19.83) | 263 (24.49) |
| 7 | “It is a woman's responsibility to avoid getting **pregnant** when a pregnancy is not desired.” | **Female** | 1105 (66.41) | 266 (15.99) | 293 (17.61) |
|  |  | **Male** | 687 (63.97) | 195 (18.16) | 192 (17.88) |
| 8 | “I would be outraged if my wife/partner asked me to use a **condom** (men) / My husband/partner would be outraged if I asked him to use a **condom** (women).” | **Female** | 858 (51.56) | 214 (12.86) | 592 (35.58) |
|  |  | **Male** | 617 (57.45) | 137 (12.76) | 320 (29.80) |
| 9 | “If a woman **cheats** on a man, it is okay for him to hit her.” | **Female** | 720 (43.27) | 254 (15.24) | 690 (41.47) |
|  |  | **Male** | 460 (42.83) | 233 (21.69) | 381 (35.47) |
| 10 | “To be a man, you need to be **tough**.” | **Female** | 597 (35.88) | 283 (17.01) | 784 (47.12) |
|  |  | **Male** | 421 (39.20) | 241 (22.44) | 412 (38.36) |
| 11 | “A man **needs other women**, even if things are fine with his wife.” | **Female** | 1022 (61.42) | 230 (13.82) | 412 (24.76) |
|  |  | **Male** | 646 (60.15) | 169 (15.74) | 259 (24.12) |
| 12 | “It is the man who **decides when** to have sex.” | **Female** | 895 (53.79) | 289 (17.37) | 480 (28.85) |
|  |  | **Male** | 556 (51.77) | 215 (20.02) | 303 (28.21) |
| 13 | “Women **need health services** more than men.” | **Female** | 1179 (70.85) | 237 (14.24) | 248 (14.90) |
|  |  | **Male** | 730 (67.97) | 193 (17.97) | 151 (14.06) |
