# Supplementary material for: Perceived gender equitable norms and previous tuberculosis testing in Malawi: A secondary analysis of a cluster-based prevalence survey
Source: PLOS Glob Public Health. 2026 Feb 12;6(2):e0004620. doi: 10.1371/journal.pgph.0004620 (PMC12900314; doi:10.1371/journal.pgph.0004620)
Supplement: S3 Table — (DOCX) [file pgph.0004620.s005.docx]

**S3 Table: Rotated Factor Loadings and CFA Goodness of Fit Statistics***

| **a. GEMS Items** | **Model 1: Two factor model (10 items)** | | **Uniqueness** |
| --- | --- | --- | --- |
|  | Sexual Autonomy and Decision-Making | Violence and Physical Toughness |  |
| It is the man who decides when to have sex | 0.423 |  | 0.688 |
| Men need sex more than women do | 0.739 |  | 0.462 |
| Men don't talk about sex, they just do it | 0.745 |  | 0.447 |
| Men are always ready to have sex | 0.738 |  | 0.435 |
| It is a woman's responsibility to avoid getting pregnant when a pregnancy is not desired | 0.462 |  | 0.707 |
| A man needs other women, even if things are fine with his wife | 0.330 |  | 0.650 |
| If a woman cheats on a man, it is okay for him to hit her |  | 0.680 | 0.703 |
| To be a man, you need to be tough |  | 0.754 | 0.730 |
| There are times when a woman deserves to be beaten |  | 0.363 | 0.683 |
| **b. Fit indices (Robust)** | | | |
| Chi 2 (degrees of freedom) | <0.001 (26) | | |
| Comparative fit index (CFI) | 0.936 | | |
| Tucker-Lewis Index (TLI) | 0.911 | | |
| Root Mean Square Error of Approximation (RMSEA) | 0.050 | | |
| **c. Factor correlations** | | | |
| Sexual Factors | 1.00 |  | |
| Violence Factors | 0.884 | 1.00 | |
