# Supplementary material for: Perceived gender equitable norms and previous tuberculosis testing in Malawi: A secondary analysis of a cluster-based prevalence survey
Source: PLOS Glob Public Health. 2026 Feb 12;6(2):e0004620. doi: 10.1371/journal.pgph.0004620 (PMC12900314; doi:10.1371/journal.pgph.0004620)
Supplement: S4 Table — (DOCX) [file pgph.0004620.s006.docx]

**S4_Table: Exploratory Obliquely Rotated Factor Loadings and Unique Variances (items 1, 8 and 13 excluded)**

| **GEMS Description** | **Factor 1** | **Factor 2** | **Uniqueness*** |
| --- | --- | --- | --- |
| There are times when a woman deserves to be beaten | 0.234 | **0.365** | 0.688 |
| Men need sex more than women do | **0.719** | 0.010 | 0.472 |
| Men don't talk about sex, they just do it | **0.750** | -0.001 | 0.440 |
| Men are always ready to have sex | **0.738** | 0.020 | 0.433 |
| It is a woman's responsibility to avoid getting pregnant when a pregnancy is not desired | **0.442** | 0.113 | 0.718 |
| If a woman cheats on a man, it is okay for him to hit her | 0.009 | **0.673** | 0.538 |
| To be a man, you need to be tough | -0.022 | **0.744** | 0.470 |
| A man needs other women, even if things are fine with his wife | **0.339** | 0.305 | 0.642 |
| It is the man who decides when to have sex | **0.422** | 0.345 | 0.491 |
