# Supplementary material for: Perceived gender equitable norms and previous tuberculosis testing in Malawi: A secondary analysis of a cluster-based prevalence survey
Source: PLOS Glob Public Health. 2026 Feb 12;6(2):e0004620. doi: 10.1371/journal.pgph.0004620 (PMC12900314; doi:10.1371/journal.pgph.0004620)
Supplement: S5 Table — (DOCX) [file pgph.0004620.s007.docx]

**S5 Table: Multivariable Regression Analysis Final Output**

|  | **Characteristic** | | **Adjusted OR** | **Wald Test p-value** |
| --- | --- | --- | --- | --- |
| Principal Association | GEMS Composite Score | | 1.11 (0.87-1.43) | 0.396 |
| **Covariates** | | | | |
| Sex | Female | | 1.00 (reference) | 0.146 |
|  | Male | | 1.27 (0.92-1.78) |  |
| HIV Status | Negative | | 1.00 (reference) | <0.001 |
|  | Positive | | 2.69 (1.99-3.64) |  |
| Age Group | 17-24 | | 1.00 (reference) | ---------- |
|  | 25-34 | | 1.56 (0.92-2.67) | 0.101 |
|  | 35-44 | | 3.16 (1.86-5.38) | <0.001 |
|  | 45-54 | | 3.41 (1.90-6.12) | <0.001 |
|  | ≥55 | | 5.58 (3.09-10.10) | <0.001 |
| HIV Testing History | No | | 1.00 (reference) | ---------- |
|  | Yes | | 2.75 (1.54-5.07) | <0.001 |
| Wealth Quantile | Step 1 | | 1.00 (reference) | ---------- |
|  | Step 2 | | 1.03 (0.60-1.77) | 0.921 |
|  | Step 3 | | 1.42 (0.86-2.37) | 0.186 |
|  | Step 4 | | 1.67 (0.95-2.93) | 0.073 |
|  | Step 5 | | 1.40 (0.60-3.24) | 0.438 |
|  | Step 6 | | 0.36 (0.04-2.94) | 0.340 |
| Education | Never attended School or not completed primary | | 1.00 (Reference) | ---------------- |
|  | Primary school leaving certificate | | 1.30 (0.91-1.86) | 0.151 |
|  | Junior certificate of education | | 0.92 (0.60-1.40) | 0.687 |
|  | Secondary with MSCE | | 1.51 (1.01-2.26) | 0.044 |
|  | Higher Education | | 1.36 (0.76-2.41) | 0.301 |
| Employment | Paid Employee | | 1.00 (Reference) | ---------------- |
|  | Piece work (Ganyu) | | 0.88 (0.51-1.52) | 0.723 |
|  | Paid domestic worker | | 1.71 (0.41-7.09) | 0.508 |
|  | Self-Employed | | 0.91 (0.63-1.32) | 0.718 |
|  | Unemployed | | 0.70 (0.46-1.05) | 0.154 |
|  | Student | | 4.62 (0.67-30.59) | 0.085 |
|  | Other | | 0.49 (0.16-1.50) | 0.215 |
| Lost spouse to death | No | | 1.00 (Reference) | ---------------- |
|  | Yes | | 1.63 (1.08-2.46) | 0.020 |
| **Reliability Check Output** | | | | |
| Rho (p-value) | | <0.001 (p=0.494) | | |
