# Supplementary material for: Perceived gender equitable norms and previous tuberculosis testing in Malawi: A secondary analysis of a cluster-based prevalence survey
Source: PLOS Glob Public Health. 2026 Feb 12;6(2):e0004620. doi: 10.1371/journal.pgph.0004620 (PMC12900314; doi:10.1371/journal.pgph.0004620)
Supplement: S6 Table — (DOCX) [file pgph.0004620.s008.docx]

**S6 Table: Interaction Model Coefficients**

| **Model Coefficient** | **Estimate** | **Standard Error** | **Z-Score** | **P-Value** |
| --- | --- | --- | --- | --- |
| **Intercept** | -3.028 | 0.194 | -15.586 | <0.001 |
| **GEMS Score** | -0.122 | 0.360 | -0.338 | 0.735 |
| **Sex** | -0.679 | 0.310 | -2.186 | 0.029 |
| **Age** | 0.032 | 0.005 | 6.480 | <0.001 |
| **GEMS Score : Sex** | 0.587 | 0.592 | 0.991 | 0.322 |
| **GEMS Score : Age** | 0.002 | 0.009 | 0.250 | 0.802 |
| **Sex : Age** | 0.021 | 0.007 | 2.843 | 0.004 |
| **GEMS Score : Sex : Age** | -0.007 | 0.014 | -0.509 | 0.611 |
