# Supplementary material for: Perceived gender equitable norms and previous tuberculosis testing in Malawi: A secondary analysis of a cluster-based prevalence survey
Source: PLOS Glob Public Health. 2026 Feb 12;6(2):e0004620. doi: 10.1371/journal.pgph.0004620 (PMC12900314; doi:10.1371/journal.pgph.0004620)
Supplement: Attachment — Submitted filename: Response to Reviewers.pdf [file pgph.0004620.s009.pdf]

Elizabeth Di Giacomo
Department of Infectious Disease Epidemiology
London School of Hygiene & Tropical Medicine
Keppel Street, London, WC1E 7HT
United Kingdom

Phone: +1 (416) 831-7708
Email: edigiac3@jh.edu

LONDON SCHOOL of HYGIENE &TROPICAL MEDICINE

Editorial Board
PLOS Global Public Health

18 June 2025

Dear Editor:

It is with pleasure that we submit our revised manuscript, *"Perceived Gender Equitable Norms and Previous Tuberculosis Testing in Malawi: A Secondary Analysis of a Cluster-based Prevalence Survey"*, for consideration for publication in PLOS Global Public Health.

This study addresses a critical gap in global TB research: the role of gender norms in shaping testing behavior, particularly among men—a population that continues to face disproportionately high burdens of TB and underutilization of services. Drawing on data from a large urban TB prevalence survey in Malawi and using a modified version of the Gender Equitable Men Scale (GEMS), we explored the association between perceived gender norms and self-reported TB testing. While we found no overall association, we observed patterns among men that varied by age and suggested that gender norm perceptions may interact with health-seeking behaviors in complex and dynamic ways.

In response to the helpful and thoughtful comments from the reviewers, we have revised the manuscript substantially to improve clarity, expand the theoretical grounding, and strengthen the interpretation of key findings. We have also provided further methodological detail, clarified analytical decisions (e.g., item weighting and factor analysis structure), and more fully contextualized our results within the limitations of cross-sectional survey data. A point-by-point response to all reviewer comments is included on the following pages.

Please contact us if any further clarification or additional information is needed. Thank you for your further consideration.

With regards,

Elizabeth Di Giacomo

Elizabeth Di Giacomo (on behalf of authors)

Reviewer #1:

1. The rationale for hypothesizing that more equitable gender attitudes would correlate with higher TB testing, particularly among men, is compelling but somewhat underdeveloped. Consider expanding the theoretical framework to include evidence from similar health behaviors (e.g., HIV testing) and gender-transformative health models in Sub-Saharan Africa.

Response: Thank you for this suggestion. We have expanded the introduction to more clearly link gender equitable attitudes with similar health behaviours in the region, including high-risk HIV behaviours and intimate partner violence. These examples highlight the possibility that more positive gender equitable norms might lead to earlier care seeking, and potentially higher rates of TB testing. We now reference gender-transformative frameworks applied in sub-Saharan Africa and cite relevant literature (in lines 42 to 44 and 59 to 62):

"*Studies are beginning to uncover the specific challenges men face when seeking TB treatment, with recent work exploring the perceptions of masculinity and stigma surrounding TB among men (8, 9)*."

"*Gender transformative interventions intended to improve health behaviours have previously demonstrated success in reducing intimate partner violence and HIV risk in African settings (17–19) This provides compelling evidence that gender norms may relate closely with TB testing behaviours and should be intentionally evaluated. We hypothesized that masculine expectations that affect health behaviours may be linked to attitudes towards gender norms, and that more equitable gender perceptions may be associated with improved access to TB services*."

2. The direction of association—where less gender-equitable views among men are linked to higher TB testing—deserves deeper interpretation. The current discussion hypothesizes that this may reflect riskier behaviors (e.g., smoking, alcohol use), but this claim is not empirically explored in the dataset. If data on behavioral risk factors exist in the parent study, consider incorporating them. Otherwise, clearly flag this as a hypothesis and discuss its implications cautiously.

Response: We agree this requires clarification. We have elaborated on this point in the discussion, emphasizing that this is a hypothesized mechanism potentially linked to unmeasured behavioral risk factors. We have clarified that these data were not collected as part of the study extended questionnaire and flagged this as a hypothesis warranting further exploration in future studies (in lines 341-343):

"*We hypothesise that the association between less gender-equitable attitudes and TB testing history may be related to certain risk behaviours that increase the probability of contracting TB. In turn, this may increase their likelihood of being tested. As these behavioural factors were not captured in the extended questionnaire, our interpretation remains speculative and should be further investigated in future studies*."

3. The finding that GEMS scores were not associated with TB testing among women, while they were among men, is interesting and should be emphasized further in the discussion. How might gender norms affect men and women differently in health-seeking? This distinction may also have implications for designing gender-transformative interventions.

Response: We appreciate this insight and have added content to the discussion emphasizing how gender norms may affect health-seeking behaviors differently for men and women, with particular reference to differences in care access norms and structural expectations. These expectations refer to the gendered organization of health systems and social roles—for example, the expectation that women will routinely engage with health services for reproductive or caregiving purposes, while men are often excluded from such pathways or perceived as less appropriate users of routine care. We have revised the discussion accordingly (in lines 396 to 399, and lines 409-413):

"While more nuanced research into these gendered perspectives in the context of TB are needed, our current understandings of the normative constraints that men experience around vulnerability, illness, and help-seeking show a need for gender-responsiveness in TB prevention and care." "While gender-specific and gender-sensitive services designed to reach men are needed to improve access to diagnosis and treatment, our work suggests an association between attitudes towards gender equitable norms and TB testing behaviours. These findings highlight the need for more comprehensive, gender-transformative strategies that seek to shift gender norms and roles in society, reducing men's excess risk of TB and enabling them to access care services."

4. The limitations section is thorough, but could be strengthened by reflecting on the challenge of measuring dynamic social norms (like gender attitudes) with cross-sectional tools. Readers would benefit from a more explicit reflection on how temporality and context may shape GEMS responses and TB testing behavior differently.

Response: We agree and have revised the limitations section to address this explicitly, noting the temporality challenges of using GEMS in a cross-sectional design and the potential for context-dependent interpretation of norm-based items. We also suggest more validation of the GEMS tool is needed against prospectively measured health outcomes, such as treatment access, morbidity, mortality, and quality of life (in lines 375-378):

"Measuring GEMS scores at a single point in time and TB testing retrospectively services as a limitation, as attitudes towards both may have evolved over time. This calls for more data on how gender norms held in these settings may change over time, potentially through repeated surveys or longitudinal study designs relating gender norms to health outcomes; analysis would benefit from further detail on the temporal alignment of individuals' TB testing with individual and societal shifts in gender norms. Finally, possibilities of accurate exposure ascertainment should be critically evaluated. Further validation work is needed for the GEMS tool against prospectively measured health outcomes such as morbidity, mortality, treatment access, and quality of life. As a complex and subjective social construct, questions surrounding how to best measure gender in this population and setting should be topics of ongoing discussion."

5. The authors mention using both exploratory and confirmatory factor analysis for the GEMS tool. Please clarify whether the same dataset was used for both, and whether split-sample validation was considered. Also, indicate the rationale for item exclusion thresholds (uniqueness > 0.70).

Response: We thank the reviewer for pointing this out. We have now specified that both analyses were conducted on the same dataset due to limited sample size. We note this as a limitation and describe the threshold of uniqueness >0.70 as a conservative cut-off used in similar psychometric work (in lines 123 to 124, and 149 to 150):

"Any items with a uniqueness value >0.70 were excluded. This recommendation is commonly used as a conservative indicator of low shared variance with other items (29–31)."

"Subsequently, factor scores were predicted using regression methods from the standardized SEM model (35), and merged into one composite score with equal weighting across all items to form the primary exposure variable for the remainder of the analysis. Higher composite scores can be interpreted as more inequitable perspectives of gender norms, and vice versa. EFA and CFA were conducted on the same dataset due to limited sample size, which may increase the potential for model overfitting."

Reviewer #2:

1. Composite GEMS score and item weighting: Please clarify whether the items used to construct the composite GEMS score were weighted.

Response: Thank you. We have clarified that the final composite GEMS score was created by averaging factor scores across retained domains with equal weighting, and we discuss the rationale for this approach in the methods section (in lines 146 and 147):

"Subsequently, factor scores were predicted using regression methods from the standardized SEM model (35), and merged into one composite score with equal weighting across all items to form the primary exposure variable for the remainder of the analysis. Higher composite scores can be interpreted as more inequitable perspectives of gender norms, and vice versa."

2. Urban context and generalizability: Clarify the setting and consider implications for generalizability.

Response: We agree this is important and have strengthened the discussion to reflect the urban nature of Blantyre and its implications for applicability in other urban settings, and conversely in rural settings (in lines 368-373):

"It may be worthwhile to evaluate this association in a rural setting, given that this study included only residents within the urban city of Blantyre. Important cultural differences may exist between rural and urban settings that change the nature of perceptions of gender norms as well as health access behaviours so the generalizability of these findings to rural contexts may be limited. Future work may consider this relationship in global settings, such as Asian and South American regions."

3. Implications for community- and policy-level interventions: Expand discussion of how findings could shape policy and community-level TB strategies.

Response: Thank you for this comment. We have expanded our discussion to include how findings could shape policy and community-level TB strategies (in lines 399-408):

"Community and policy level interventions may include structural efforts to shift gender equitable norms, potentially increasing willingness to seek TB testing. These interventions would target the upstream determinants of TB risks such as social norms, education, and health system design to improve gender-equitable norms and improve willingness to test for TB. For example, policies that support gender-transformative education campaigns or involve men in community-based health promotion may help challenge norms that discourage care seeking. Adapting health services by offering clinic hours amenable to men's work schedules can address practical barriers which disproportionately affect men. Tailoring both messaging and service delivery to account for gender-specific needs might improve engagement with TB testing and care."
