# Supplementary material for: Perceived gender equitable norms and previous tuberculosis testing in Malawi: A secondary analysis of a cluster-based prevalence survey
Source: PLOS Glob Public Health. 2026 Feb 12;6(2):e0004620. doi: 10.1371/journal.pgph.0004620 (PMC12900314; doi:10.1371/journal.pgph.0004620)
Supplement: Attachment — Submitted filename: Response_to_Reviewers_auresp_2.pdf [file pgph.0004620.s011.pdf]

Elizabeth Di Giacomo
Department of Infectious Disease Epidemiology
London School of Hygiene & Tropical Medicine
Keppel Street, London, WC1E 7HT
United Kingdom

Phone: +1 (416) 831-7708
Email: edigiac3@jh.edu

LONDON SCHOOL *of* HYGIENE &TROPICAL MEDICINE

Editorial Board

PLOS Global Public Health

29 August 2025

Dear Editor:

It is with pleasure that we submit our revised manuscript, *"Perceived Gender Equitable Norms and Previous Tuberculosis Testing in Malawi: A Secondary Analysis of a Cluster-based Prevalence Survey"*, for further consideration for publication in PLOS Global Public Health.

This study addresses a critical gap in global TB research: the role of gender norms in shaping testing behavior, particularly among men—a population that continues to face disproportionately high burdens of TB and underutilization of services. Drawing on data from a large urban TB prevalence survey in Malawi and using a modified version of the Gender Equitable Men Scale (GEMS), we explored the association between perceived gender norms and self-reported TB testing. While we found no overall association, patterns among men varied by age and suggested that gender norm perceptions may interact with health-seeking behaviors in complex and context-specific ways.

In response to the insightful feedback from the reviewers, we have further expanded the discussion to strengthen the theoretical framing, deepen the interpretation of null results, and address the practical implications of our findings for TB program design. We have also provided additional commentary on non-linear patterns in TB testing prevalence by age category, clarified methodological details, and articulated more clearly how this work contributes to the broader literature on gender and TB. A detailed, point-by-point response to all reviewer comments is included on the following pages.

Please contact us if any further clarification or additional information is needed. Thank you for your continued consideration.

With regards,

Elizabeth Di Giacomo (on behalf of authors)

Reviewer #3: This is a promising and well-designed study, but the manuscript would benefit from deeper theoretical engagement with the findings, clearer discussion of null results, and a stronger articulation of how this work contributes to the broader literature on gender and TB. Enhancing these aspects will improve the manuscript's impact and clarity.

Response: Thank you for this suggestion. We have re-examined the Discussion section of the manuscript and substantially revised it to more clearly discuss the study findings and articulate how this work contributes to the broader literature on gender and TB.

Reviewer #4: This study is well designed and executed. The topic and the findings are relevant to the field of TB prevention and control and global health more broadly.
The discussion does not adequately address two key issues, however.
The association noted between TB testing and age is not surprising since the variable is measured as "ever tested." The authors note that the association is not linear, but provide not further commentary on variation in testing status by age category. If they want to discuss this finding, they should include some commentary on whether the data suggest that any more complex relationship between age and "ever tested" exists than the simple passage of time.

More significantly, the discussion should reflect more on the practical implications of the lack of an association between GEMS score and testing status. The conclusions state merely that more nuanced research is needed. If, in fact, there is no association between gender equitable attitudes and health service seeking behavior, this would have implications for program design. These findings may indicate that the topic merits more research. At the same time it may also mean that, in fact, gender norms do not have the hypothesized effect on health service seeking behavior and should not be considered a lever for that particular change. The discussion should reflect a consideration of this possibility.

Response: We appreciate the suggestion to expand discussion of non-linear age associations and practical implications of our findings. We now comment on the potential explanations for non-linear variation in TB testing prevalence across age categories and highlight how generational differences or historical changes in service access may contribute to these patterns (Lines 394-398):

"However, age and greater cumulative opportunity for testing may only partially explain our results, as assessment of linear trend signaled variation across age categories that was not strictly linear. This may indicate that factors other than simple time at risk – such as generational differences or historical changes in service accessibility – may also shape testing behaviour across age groups."

We have also expanded discussion of to add more explicit consideration of the practical implications of the null association between GEMS score and TB testing, including the possibility that gender norms may not serve as the hypothesized driver of TB care-seeking in this context and the resulting implications for program design throughout the Discussion section of the manuscript.

In-text comment 1
Original text: "An estimated 10.8 million people fell ill with TB in 2023, with 1.25 million deaths, making it the deadliest infectious disease among adults."
Reviewer comment: Reference? Across globe??

Response: We have revised the text to state that these are global estimates and we have added a reference to the WHO Global TB Report (Lines 26-27), as follows:

"An estimated 10.8 million people fell ill with TB globally in 2023, with 1.25 million deaths, making it the deadliest infectious disease among adults (2)."

In-text comment 2
Original Text: "They are considered important social determinants of health (12,13)"
Reviewer comment: Doesn't seem to be at appropriate place or incomplete sentence. You have already stated importance of gender norm before

Response: We have rephrased acknowledgement of the role of gender norms as social determinants of health (Lines 47-51), as follows:

"Gender norms are considered important social determinants of health and have been defined as "a subset of social norms that relate specifically to gender differences. They are informal, deeply entrenched and widely held beliefs about gender roles, power relations, standards or expectations that govern human behaviours and practices in a particular social context and at a particular time" (11–13)"

In-text comment 3
Original text: "This provides compelling evidence that gender norms may relate closely with TB testing behaviours and should be intentionally evaluated"
Reviewer comment: Need to make strong argument. How your study is significant and contribution of this study?

Response: We have rephrased this section (Lines 51-60), as follows:

"Research has highlighted how masculine expectations of physical strength, stoicism, and control lead men to avoid admissions of illness and neglect TB symptoms until they become incapacitating, and how men deprioritise their own healthcare needs to fulfil roles as leaders and providers (5,9,14,15). This evidence associating men's healthcare decisions with masculine norms and expectations suggests gender norms may influence TB testing behaviours, but such an association has not been explored quantitatively."

In-text comment 4
Original text: We hypothesized that masculine expectations that affect health behaviours may be linked to attitudes towards gender norms, and that more equitable gender perceptions may be associated with improved access to TB services.
Reviewer comment: DIfferentiate between attitude and perception here

Response: We have standardised text throughout the manuscript to refer to "attitudes" rather than "perceptions", for example, in lines 56-59:

"We hypothesized that masculine expectations that affect health behaviours may be linked to attitudes towards gender norms, and that more equitable gender attitudes may be associated with improved access to TB services."

In-text comment 5
Original text: A better understanding of the relationship between perspectives of gender norms and previous TB testing could identify new approaches and methods to reach men, in particular, with TB diagnostic services. This could result in more efficient public health strategies to improve diagnosis and treatment of individuals with TB and ultimately reduced transmission in the fight against TB, particularly among men as an underserved population.
Reviewer comment: There are studies depicting gender differences to access to care where females have limited access than males. How you acknowledge this?

Response: Studies of patient, diagnostic, and treatment delays provide mixed evidence on gender differences in access to TB care, with some showing longer delays among women and others longer delays among men. However, these studies are at risk of recall bias, as men and women may vary in their recognition of disease onset, and survivorship bias, as studies are typically conducted among individuals who have accessed care. In most settings, empirical indicators of gaps in detection and reporting, based on prevalence and notification data, are inconsistent with more limited access among women than men. Whilst there is a need to improve access to

tuberculosis care for all individuals, the more pressing need is to understand and address men's barriers to care.

In-text comment 6
Original text: We conducted a secondary analysis of data from a community TB prevalence pre-intervention survey implemented from June 2019 to March 2020 before the active case finding cluster-randomised trial "Sustainable Community-wide Active Case Finding for Lung hEalth (SCALE)" (ISRCTN11400592) (20,21)
Reviewer comment 1: Where??
Reviewer comment 2: Mention study site

Response: We have added text to clarify that the survey was conducted in Blantyre, Malawi (Lines 99-102), as follows:

"We conducted a secondary analysis of data from a community TB prevalence pre-intervention survey in Blantyre, Malawi implemented from June 2019 to March 2020 before the active case finding cluster-randomised trial "Sustainable Community-wide Active Case Finding for Lung hEalth (SCALE)" (ISRCTN11400592) (20,21)"

In-text comment 7
Original text: For the prevalence survey, households were randomly sampled from community neighbourhood clusters, and all adults (≥18 years) were invited to participate in a questionnaire and TB screening.
Reviewer comment: Please confirm if this was questionnaire or interview schedule

Response: We have clarified that the questionnaire was administered by an interviewer (Lines 102-107), as follows:

"For the prevalence survey, households were randomly sampled from community neighbourhood clusters, and all adults (≥18 years) were invited to participate in an interviewer-administered questionnaire and TB screening. A 20% random sub-sample of participants were additionally asked to complete an extended interviewer-administered questionnaire that included questions on TB testing history, perceptions of gender equitable norms, healthcare utilization and social contacts mixing (Supplementary Figure 1). A 20% random sub-sample of participants were additionally administered an extended questionnaire that included questions on TB testing history, perceptions of gender equitable norms, healthcare utilization and social contacts mixing (Supplementary Figure 1). "

In-text comment 8
Original text: Criteria for inclusion were age of 18 years or older on the day of the interview; able to provide written or witnessed informed consent; intention to remain resident in Blantyre for at least 8 weeks following recruitment; and be willing to have radiological/microbiological screening for TB.
Reviewer comment: If this paper is based on secondary analysis why did you stated this here?

Response: While the manuscript is based on a secondary analysis, we included these details to help the reader understand the underlying study population. We have clarified the text (Lines 107-111), as follows:

"Inclusion criteria for the original study from which these data were drawn were age of 18 years or older on the day of the interview; able to provide written or witnessed informed consent; intention to remain resident in Blantyre for at least 8 weeks following recruitment; and be willing to have radiological/microbiological screening for TB."

In-text comment 9
Original text: In this secondary analysis of data from a TB prevalence survey conducted in Malawi as part of a community cluster-randomised trial, we investigated the association between perceptions of gender norms and self-reported TB testing history using a validated tool to measure gender attitudes.
Reviewer comment: "*In this secondary analysis of data from a TB prevalence survey conducted in Malawi as part of a community cluster-randomised trial, we investigated the association between perceptions of gender norms and self-reported TB testing history using a validated tool to measure gender attitudes."* Clarify.

Revised text: In this secondary analysis of data, we used data collected from a TB prevalence survey conducted in Malawi as part of a community cluster-randomised trial. We investigated the association between perceptions of gender norms and self-reported TB testing history using a validated tool to measure gender attitudes.

In-text comment 10
Original text: A better understanding of the relationship between perspectives of gender norms and previous TB testing could identify new approaches and methods to reach men, in particular, with TB diagnostic services.
Reviewer comment: Justify this clearly

Response: We have expanded our discussion of how a better understanding of the relationship between gender norms and TB testing decisions in the Discussion section to more clearly justify links between gender norms and TB testing, acknowledging the null results of the studies and discussing the difference in trends between sexes across age groups.

In-text comment 11
Original text: The primary exposure was individual perception of gender equitable norms, measured using the Gender Equitable Men Scale (GEMS) (22).
Reviewer comment: Justify use of this scale

Response: The GEMS scale was selected as a validated, standardised framework for quantifying gender-related attitudes. We have added this to the text (lines 131-133), as follows:

"The primary exposure was individual perception of gender equitable norms, measured using the Gender Equitable Men Scale (GEMS) (22), which provides a validated, standardised framework for quantifying gender attitudes."

In-text comment 12
Original text: While the full GEMS consists of 42-items with domains covering violence, sexual relationships, reproductive health and disease prevention, and domestic chores and daily life (23), the SCALE Trial employed a modified 13-item version of the scale, available in both English and Chichewa (see S1 Table)
Reviewer comment: Why did the study use the modified version?

Response: The shorter version was selected to reduce respondent burden in the context of a large prevalence survey while still capturing the core domains relevant to gender norms and health-seeking behaviours. The modified version used in this study had previously been used in Malawi to investigate gender attitudes and HIV risk (Lines 136-139):

"The scale is adaptable, and different versions have been used in a variety of settings (23). This particular adaptation was informed by previous work in Malawi investigating men's gender attitudes and HIV risk, and a peer-delivered behavioural intervention on contraceptive uptake (24)."

In-text comment 13
Original text: All participants were asked to report their HIV and antiretroviral therapy (ART) status, and were offered HIV testing. Individuals who self-reported a positive HIV test result, or ART use, or who tested positive were classified as HIV-positive.

Reviewer comment: Why only HIV status? Why other risk factors for TB were not considered for exploration?

Response: While it would have been interesting to explore other gendered risks for TB, we were limited by the data available in this secondary analysis. We have noted this (Lines 162-166), as follows:

"All participants were asked to report their HIV and antiretroviral therapy (ART) status, and were offered HIV testing. Individuals who self-reported a positive HIV test result, or ART use, or who tested positive were classified as HIV-positive. Other behavioural or clinical risk factors for TB, such as smoking, alcohol use, diabetes, or malnutrition, were not collected as part of the extended questionnaire and therefore could not be explored in this secondary data analysis."

In-text comment 14
Original text: Of the 15,897 individuals enrolled in the SCALE Trial, 2,738 participants across 72 clusters completed the extended questionnaire containing the GEMS survey (Figure S1).
Reviewer comment: Describe how they were enrolled

Response: As this is a secondary data analysis, we feel details on enrolment procedures are beyond the scope of this paper. We have referenced papers describing the original trial in the Methods section, to which readers may refer for additional details (Lines 99-102), as follows:

"We conducted a secondary analysis of data from a community TB prevalence pre-intervention survey in Blantyre, Malawi, implemented from June 2019 to March 2020 before the active case finding cluster-randomised trial "Sustainable Community-wide Active Case Finding for Lung hEalth (SCALE)" (ISRCTN11400592) (20,21)."

In-text comment 15
Original text: 15.8% (433/2,738) of the total population reported ever testing for TB (Table 1). 15.5% of women reported ever testing for TB, while 16.3% of men reported previously testing (Chi-square p=0.581).
Reviewer comment: Was their knowledge for TB assessed?

Response: Participants' knowledge of TB was not assessed in this study.

In-text comment 16

Original text: Testing proportions were lowest among the youngest age group (17-24 years) with only 14.6% reporting having ever tested.
Reviewer comment 1: Should be 18-24 years?
Reviewer comment 2: why everywhere this is 17 when the inclusion criteria defined is more than 18 years?

Response: Thank you for noting this mistake. We have corrected the text here and throughout the manuscript to recognise that the youngest age group was ages 18-24 years (lines 245-247), as follows:

"Testing proportions were lowest among the youngest age group (18-24 years) with only 14.6% reporting having ever tested."

In-text comment 17
Original text: [Table 1] Presence Of TB Symptoms (cough, night sweats, weight loss, and/or fever)
Reviewer comment: Only cough or persistent for specific duration??

Response: We have clarified that cough refers to "any cough" rather than cough of a specific duration, and revised the text in the table as follows:

"Presence Of TB Symptoms (any cough, night sweats, weight loss, and/or fever)"

In-text comment 18
Original text: For all GEMS items except for Items 3, 9 and 10 (Supplementary Table 2), the majority of men and women reported strong agreement with the statement. Internal consistency does vary with adaptation of the GEMS tool, however remained very high in the context of this study (Cronbach's alpha = 0.88).
Reviewer comment: Elaborate on the cultural appropriateness of the scale and its psychometric properties in this context. Was the scale pre-tested or validated in Malawi prior to its use in this study?

Response: The GEMS scale is adaptable and has been used in a variety of settings. The modified version used in this study had previously been used in Malawi to investigate gender attitudes and HIV risk (Lines 136-139):

"The scale is adaptable, and different versions have been used in a variety of settings (23). This particular adaptation was informed by previous work in Malawi investigating men's gender

attitudes and HIV risk, and a peer-delivered behavioural intervention on contraceptive uptake (24).”

In-text comment 19
Original text: In the logistic model that included GEMS score, TB testing history and all covariates, we did not identify a statistically significant association between participants' GEMS score and their likelihood of having previously tested for TB (OR = 1.11, 95% CI: 0.87-1.43, p=0.396) (see S5 table).
Reviewer comment: Incomplete sentence

Response: We have reviewed the text noted by the reviewer and do not believe this to be an incomplete sentence (Lines 311-314):

“In the logistic model that included GEMS score, TB testing history, and all covariates, we did not identify a statistically significant association between participants' GEMS score and their likelihood of having previously tested for TB (OR = 1.11, 95% CI: 0.87-1.43, p=0.396) (see S5 table).”

In-text comment 20
Original text: We did not detect a statistically significant association between perceptions of gender equity, as measured by GEMS, and the odds of self-reporting ever testing for TB. However, overall, we identified a potential effect of age on GEMS score 295 and the probability of previous TB testing among men. For men, those with higher GEMS scores (less gender-equitable views) were more likely to have been tested for TB across age groups. It is notable that no associations between GEMS score and previous TB testing were noted among women.
Reviewer comment: How it is across gender in that age group?

Response: The potential effect of age on GEMS score and the probability of previous TB testing was noted only among men; no associations between GEMS score and previous TB testing history were noted among women. (Lines 355-358):

“However, among men, we identified a potential effect of age on GEMS score and the probability of previous TB testing among men. For men, those with higher GEMS scores (less gender-equitable views) were more likely to have been tested for TB across age groups. It is notable that no associations between GEMS score and previous TB testing were noted among women.”

In-text comment 21
Original text: To our knowledge, this is the first study to assess the impact of perceptions of gender equity and gender norms on a TB health outcome.
Reviewer comment: Was GEMS used to assess impact on other disease outcomes?

Response: GEMS has previously been used in a variety of settings and with other disease outcomes. We discuss other uses (Lines 401-405), as follows:

"The use of the GEMS tool to assess gender attitudes has been established in the fields of HIV, reproductive health, and intimate partner violence (17–19). The strengths of the tool in this literature include a validated framework for quantifying attitudes that are often difficult to capture, and evidence that changes in GEMS scores correlate with meaningful improvements in health outcomes and reductions in gender-based violence."

In-text comment 22
Original text: Sexual factors have been cited in previous qualitative work investigating drivers of the sex differences in health service access (6). Sexual factors were mentioned in the context of a man's need for sex, and how a diagnosis of TB can impede opportunities for sexual activity in their relationship (6). Themes of physical violence were less explicitly stated in literature, however the GEMS items underpinning this latent factor are supported. The idea that toughness is inherent to masculinity is ubiquitous, and TB is understood by individuals to threaten or emasculate men (5,6).
Reviewer comment: Need to explain how these studies vary or cohere with the current study?

Response: We have clarified that these studies cohere with our findings by clarifying themes identified by latent factors in our work (Lines 365-384), as follows:

"However, the latent factors extracted from this factor analysis agree with the themes and ideas expressed in existing literature exploring the drivers of sex differences in TB health-seeking behaviours. Sexual factors have been cited in previous qualitative work investigating drivers of the sex differences in health service access (6). Sexual factors were mentioned in the context of a man's need for sex, and how a diagnosis of TB can impede opportunities for sexual activity in their relationship (6). Themes of physical violence were less explicitly stated in literature, however the GEMS items underpinning this latent factor are supported. The idea that toughness is inherent to masculinity is ubiquitous, and TB is understood by individuals to threaten or emasculate men (5,6). Our findings are broadly consistent with this qualitative literature in underscoring the salience of masculine norms – particularly toughness and control – as barriers to timely health seeking. Our study differed from the prior work in that our quantitative analysis

did not identify a clear link between gender attitudes and TB testing. This divergence may highlight how different dimensions of gender norms might operate across study designs."

In-text comment 24
Original text: The use of behavioural interventions to address gender attitudes have been previously investigated (17–19). The Intervention with Microfinance for AIDS and Gender Equity (IMAGE) study assessed the use of structural interventions, including training on gender roles, communication skills, and relationships. More progressive gender norms and a substantial reduction in intimate partner violence were reported (17). Community mobilization as an intervention has specifically been evaluated on GEMS responses, in the context of HIV in South Africa (19). The trial noted a statistically significant improvement in GEMS scores among men who received a theory-based, gender transformative intervention. This type of intervention aimed at shifting community gender norms may have far reaching effects for engagement with TB health services for men. A Cochrane review on active case finding highlighted that identification of cases is only part of the work to be done in promoting health service use for TB treatment. Engendered stigma seen in HIV is compounded on to TB and contributes to discrimination, calling for community-based interventions to improve uptake (48). As a social construct, a platform for gender transformative education may be a valuable mechanism to promote community buy-in that is essential for shifts in gender attitudes.
Reviewer comment: It's difficult to relate these studies with findings of your study. Need to show some correlation else this is is just presenting the findings from other studies. Suggesting to re-write this para discussing advantages or disadvantages of use of GEMS for specific diseases carrying gender differences or stigmatized conditions

Response: Thank you for this suggestion. We have re-written this paragraph to discuss the advantages or disadvantages of use of GEMS for specific diseases carrying gender differences or stigmatized conditions (Lines 401-415).

Revised text: The use of the GEMS tool to assess gender attitudes has been established in the fields of HIV, reproductive health, and intimate partner violence (17–19). The strengths of the tool in this literature include a validated framework for quantifying attitudes that are often difficult to capture, and evidence that changes in GEMS scores correlate with meaningful improvements in health outcomes and reductions in gender-based violence. However, GEMS has primarily been applied to conditions where gender inequity is a direct determinant of risk or service use, and its adaptation to new disease contexts requires careful consideration of cultural appropriateness and relevance of scale items. Tuberculosis shares some features with these conditions, particularly stigma and gendered barriers to care, yet also differs in that biological and structural risk factors (such as HIV status, poverty, or occupational exposure) play a large role. The application of GEMS to TB therefore provides an opportunity to explore whether

gender attitudes extend their influence on this domain, while also highlighting the need for additional research to determine its validity and utility in the context of TB.

In-text comment 25
Original text: Community and policy level interventions may include structural efforts to shift gender equitable norms, potentially increasing willingness to seek TB testing. These interventions would target the upstream determinants of TB risks such as social norms, education, and health system design to improve gender-equitable norms and improve willingness to test for TB. For example, policies that support gender-transformative education campaigns or involve men in community-based health promotion may help challenge norms that discourage care seeking. Adapting health services by offering clinic hours amenable to men's work schedules can address practical barriers which disproportionately affect men. Tailoring both messaging and service delivery to account for gender-specific needs might improve engagement with TB testing and care.
While gender-specific and gender-sensitive services designed to reach men are needed to improve access to diagnosis and treatment, our work suggests an association between attitudes towards gender equitable norms and TB testing behaviours.
Reviewer comment: Need to add a stronger articulation of how this work contributes to the broader literature on gender and TB. Enhancing these aspects will improve the manuscript's impact and clarity

Response: We have re-examined the Discussion section of the manuscript and revised it to more clearly discuss the study findings and articulate how this work contributes to the broader literature on gender and TB.

In-text comment 26
Original text: These findings highlight the need for more comprehensive, gender-transformative strategies that seek to shift gender norms and roles in society, reducing men's excess risk of TB and enabling them to access care services.
Reviewer comment: Findings are not coherent to this

Response: We have expanded discussion of to add more explicit consideration of the practical implications of the null association between GEMS score and TB testing, including the possibility that gender norms may not serve as the hypothesized driver of TB care-seeking in this context and the resulting implications for program design throughout the Discussion section.
