# Supplementary material for: Perceived gender equitable norms and previous tuberculosis testing in Malawi: A secondary analysis of a cluster-based prevalence survey
Source: PLOS Glob Public Health. 2026 Feb 12;6(2):e0004620. doi: 10.1371/journal.pgph.0004620 (PMC12900314; doi:10.1371/journal.pgph.0004620)
Supplement: Attachment — Submitted filename: Response_to_Reviewers_auresp_3.pdf [file pgph.0004620.s013.pdf]

Elizabeth Di Giacomo
Department of Infectious Disease Epidemiology
London School of Hygiene & Tropical Medicine
Keppel Street, London, WC1E 7HT
United Kingdom
Phone: +1 (416) 831-7708

Email: edigiac3@jh.edu

LONDON SCHOOL of HYGIENE &TROPICAL MEDICINE

Editorial Board
PLOS Global Public Health

10 December 2025

Dear Editor:

It is with pleasure that we submit our revised manuscript, *"Perceived Gender Equitable Norms and Previous Tuberculosis Testing in Malawi: A Secondary Analysis of a Cluster-based Prevalence Survey"*, for further consideration for publication in PLOS Global Public Health.

This study addresses a critical gap in global TB research: the role of gender norms in shaping testing behavior, particularly among men—a population that continues to face disproportionately high burdens of TB and underutilization of services. Drawing on data from a large urban TB prevalence survey in Malawi and using a modified version of the Gender Equitable Men Scale (GEMS), we explored the association between perceived gender norms and self-reported TB testing. While we found no overall association, patterns among men varied by age and suggested that gender norm perceptions may interact with health-seeking behaviors in complex and context-specific ways.

In response to the feedback from the reviewers, we have added recent gender-TB papers to strengthen positioning, rephrased figure captions to be descriptive, and ensured consistent phrasing when discussing the implications of our findings. A detailed, point-by-point response to all reviewer comments is included on the following pages.

Please contact us if any further clarification or additional information is needed. Thank you for your continued consideration.

With regards,

Elizabeth Di Giacomo

Elizabeth Di Giacomo (on behalf of authors)

Journal Requirements:

1. Please provide a detailed online Financial Disclosure statement. This is published with the article. It must therefore be completed in full sentences and contain the exact wording you wish to be published.

a) State the initials, alongside each funding source, of each author to receive each grant. For example: “This work was supported by the National Institutes of Health (####### to AM; ###### to CJ) and the National Science Foundation (###### to AM).”

This statement is made on Page 1 of the manuscript:
“Funding statement: PM, KCH, and ELC are supported by the UK FCDO (“Leaving no-one behind: transforming gendered pathways to health for TB”). KCH is also supported by the US National Institutes of Health (R-202309-71190). ESN is funded by the Polio Research Committee (WHO; ref: 1432457-1).

This research has been partially funded by UK aid from the UK government (to PM, ELC and KCH); however the views expressed do not necessarily reflect the UK government’s official policies.”

b) State what role the funders took in the study. If the funders had no role in your study, please state: “The funders had no role in study design, data collection and analysis, decision to publish, or preparation of the manuscript.”

We have added a statement on the role of funders in our study to our Funding Statement on Page 1:

“The funders had no role in study design, data collection and analysis, decision to publish, or preparation of the manuscript.”

2. Please ensure that the funders and grant numbers match between the Financial Disclosure field and the Funding Information tab in your submission form. Note that the funders must be provided in the same order in both places as well.

We have confirmed that the funders and grant numbers match between the Financial Disclosure field and the Funding Informations tab.

3. Please upload your main article file as a .doc, .docx or .rtf file.

We have uploaded the main article file as a .docx file.

4. Please provide separate figure files in .tif or .eps format only and remove any figures embedded in your manuscript file. Please also ensure that all files are under our size limit of 10MB. Please leave the figure captions in the manuscript.

We have removed these files and uploaded them separately as .tif files that meet the Journal’s guidelines. The captions have been left in the manuscript.

5. We notice that your supplementary figures and tables are included in the manuscript file. Please remove them and upload them with the file type 'Supporting Information'. Please ensure that each Supporting Information file has a legend listed in the manuscript before or after the references list.

We have now included them in a separate file as per your guidance, with a legend in the manuscript after the references list.

6. If the reviewer comments include a recommendation to cite specific previously published works, please review and evaluate these publications to determine whether they are relevant and should be cited. There is no requirement to cite these works unless the editor has indicated otherwise.

We have only included citations which we have reviewed and evaluated to be relevant to the manuscript.

7. Please review your reference list to ensure that it is complete and correct. If you have cited papers that have been retracted, please include the rationale for doing so in the manuscript text, or remove these references and replace them with relevant current references. Any changes to the reference list should be mentioned in the rebuttal letter that accompanies your revised manuscript. If you need to cite a retracted article, indicate the article’s retracted status in the References list and also include a citation and full reference for the retraction notice.

We have reviewed the reference list to confirm that it is complete and correct.

Reviewer Comments:

Reviewer #3: There are minor issues to be addressed:-

Phrases like "our work suggests" & "demonstrates" need to be consistent

We have replaced the word “demonstrate” with “suggest” in line 333 for consistency:

“Our results also suggest an increase in history of previous TB testing with age and HIV status, which is consistent with accumulated testing opportunities over the life course and increased opportunities for TB testing for individuals in HIV care (42-44).

Ensure captions are descriptive (e.g. figure 2)

We have expanded captions to be more descriptive, as follows:

"Figure 1: Responses to each item on the adapted Gender Equitable Men Scale by sex"

"Figure 2: Predicted probability (with 95% confidence intervals) of ever testing for TB by GEMS score, age, sex, and HIV status"

References: Some recent Gender-TB papers could be added to strengthen positioning.

We have added more recent papers on gender and TB, as follows:

(line 302) "The potential association between less gender-equitable attitudes and increased likelihood of TB testing history among men could reflect increased risk behaviours that increase the probability of developing TB and, in turn, being tested."

(line 329) "Whereas gender norms push men towards stoicism and self-reliance (5,6)..."

(line 364) "These may include factors more closely linked with documented structural access barriers, perceptions of symptom severity, or the harmonization of routine care with TB care."
